# Postmortem detection and histopathological features of canine spirocercosis-induced putative esophageal chondrosarcoma

**DOI:** 10.14202/vetworld.2018.1376-1379

**Published:** 2018-10-03

**Authors:** H. M. Suranji Wijekoon, D. M. S. Munasinghe, K. A. N. Wijayawardhane, H. M. H. S. Ariyarathna, Neil Horadagoda, Jayanthe Rajapakse, D. D. Niranjala De Silva

**Affiliations:** 1 Department of Veterinary Pathobiology, Division of Parasitology, Faculty of Veterinary Medicine and Animal Science, University of Peradeniya, Sri Lanka; 2 Department of Veterinary Clinical Sciences, Veterinary Teaching Hospital, Faculty of Veterinary Medicine and Animal Science, University of Peradeniya, Sri Lanka; 3 Department of Basic Veterinary Sciences, Faculty of Veterinary Medicine and Animal Science, University of Peradeniya, Sri Lanka; 4 University Veterinary Teaching Hospital Camden, University of Sydney, 410 Werombi Road, Camden, NSW 2570, Australia

**Keywords:** chondrosarcoma, dog, esophagus, histopathology, *Spirocerca lupi*, spirocercosis

## Abstract

**Aim:**

The objective of this study was to describe and characterize the postmortem and histopathological findings of putative esophageal chondrosarcoma associated with *Spirocerca lupi*.

**Materials and Methods:**

Spirocerca-associated esophageal nodules were collected from 54 dogs at postmortem examination and were stained with hematoxylin and eosin. Of the cases examined, 15 were selected randomly for further investigation, of which 11 were classified as non-neoplastic nodules while 4 had changes reflecting a neoplastic process.

**Results:**

In all four neoplastic cases, the wall of the esophageal nodules contained islands and nests of highly proliferative atypical chondroblasts within a cartilaginous matrix. However, there was no statistically significant association between gender (p=0.228), age (p=0.568), and breeds (p>0.05) with the occurrence of spirocercosis. Moreover, all esophageal nodules identified were located near the caudal segment, and their diameters ranged from 1 to 6 cm (4.7±1.5 cm). A number of worms in each nodule varied from 5 to 25 (11.3±5).

**Conclusion:**

Histopathology and cytology revealed that the wall of the esophageal nodules contained islands and nests of highly proliferative atypical chondroblasts within a cartilaginous matrix, a rare finding, and clinical challenge in spirocercosis.

## Introduction

*Spirocerca lupi* is a nematode belonging to the order of *Spirurida* that is responsible for spirocercosis. *S. lupi* is a globally distributed nematode but commonly found in dogs in tropical and subtropical countries [1]. Spirocercosis is a debilitating disease and may even cause acute death due to rupture of an aortic aneurysm but is often presented with chronic clinical manifestations resulting from esophageal nodular granuloma, malignancies, and secondary pulmonary osteoarthropathy [2]. The clinical signs of spirocercosis vary on the stage of the disease, possible complications, and presence of aberrant migrations. Early migration of larvae through the gastric mucosa causes acute onset of vomiting [1]. An uncomplicated infection may be subclinical or show evidence of vomiting/regurgitation, weight loss, and dysphagia due to the development of distal esophageal nodules [3].

At present, spirocercosis has generated significant interest among oncologists and pathologists in the world due to hyperplastic and neoplastic changes that arise secondary to *S. lupi* infection. However, esophageal tumors are rare in dogs and represent only 0.5% of canine neoplasms [4]. *S. lupi* is distributed worldwide, especially in warm climatic regions. Cases of canine spirocercosis commonly seen in practice since it first reported in Sri Lanka in the 1960s [5].

However, among the few reports of *S. lupi* prevalence data in Sri Lanka, there is no literature on the comprehensive histopathological study on *S. lupi*-induced esophageal nodules. To the best of our knowledge, this is the first detailed histological study used to diagnose the spirocercosis-induced putative esophageal chondrosarcoma on dogs at autopsy.

## Materials and Methods

### Ethical approval

This experiment was carried out in accordance with the guidelines for animal experimentation of the Faculty of Veterinary Medicine and Animal Sciences, University of Peradeniya, Sri Lanka.

### Samples

A cross-sectional study on dogs died suspecting of *S. lupi* and other dog cadavers which were subjected to autopsy examinations were used to determine the prevalence of spirocercosis in dogs at Veterinary Teaching Hospital (VTH), Peradeniya, Sri Lanka, between August 2013 and February 2014. However, due to the chronic nature of spirocercosis, dogs were diagnosed in advanced disease stages, which are associated with severe detectable clinical signsas mentioned in Table-1 together with the aids of fecal flotation, radiography, and endoscopy. On obtaining owner’s consent, cadavers were subjected to an autopsy within 24 h of death. The medical history of each dog of spirocercosis suspected or non-suspected was documented including age, sex, and breed.

**Table-1:** Clinical signs of Spirocerca positive dogs (n=54).

Clinical signs	Number of dogs (%)
Gastrointestinal tract	n=18 (33%)
Vomiting, regurgitation	9
Hypersalivation	3
Dysphagia	10
Hematemesis	4
Respiratory	n=16 (29%)
Dyspnea	9
Cough	12
Abnormal respiratory sounds	13
Neurological	n=4 (7%)
Paralysis	4
Musculoskeletal	n=2 (3%)
Back pain	2
Weakness/lethargy	2
Other	
Anorexia	10
Weakness	5
Weight loss	14
Generalized lymphadenopathy	4
Acute death	6
No clinical signs	n=6 (11%)

Some individuals have shown more than one symptoms

Tissue samples were collected from esophageal nodules, preserved in 10% neutral buffered formalin for subsequent histopathological processing. The information relevant to the lesion was recorded, including size and location of the nodules, macroscopic view of the nodular cut sections, and a number of worms in the nodules. Worms and eggs from each suspected nodule were microscopically observed for further confirmation of *S. lupi*.

### Histopathology

Histopathological study was done using 15 esophageal nodular sections collected at autopsy. Around 2 cm^2^ tissue sections from lesion associated with *S. lupi* were excised from the esophagus. Tissue processing was done in an automated processor (TP 1020, Leica, Germany) and sectioned (4-5 µm) using a manual rotary microtome (RM2235, Leica, Germany). After deparaffinization, sections were stained with hematoxylin and eosin (H and E) and Van Gieson stain (Sigma, St. Louise, MO, USA). The stained tissue sections were examined using a research microscope (Accu-scope 3000 series, Hicksville, NY, USA).

### Statistical analysis

All recorded data related to signalment were analyzed by Chi-square (χ^2^) test using the SPSS software (ver. 07 for Windows; SPSS Inc., Chicago, IL, USA). Statistical significances were achieved when p<0.05.

## Results and Discussion

A total of 163 cadavers of *S. lupi* suspected and non-suspected dogs were examined at VTH during the 6 months period of study in which 54 dogs (33.1%) were diagnosed as spirocercosis with different degrees of severity. In this study, the most prominent clinical signs presented by *S. lupi*-infected dogs were retrospectively evaluated and identified as vomiting, regurgitation, weight loss, salivation, dysphagia, odynophagia, and lethargy. The predominant clinical signs of dogs with esophageal tumors mimicked those of dogs suffering from partial esophageal obstruction, including those with spirocercosis-induced granulomas. Percentage of gastrointestinal system associated clinical manifestations (33%) is highly evident among spirocercosis dogs according to our retrospective study (Table-1). This finding is consistent with other reports which emphasized regurgitation and/or vomiting as common clinical signs in dogs with spirocercosis [6-8].

**Figure-1:**
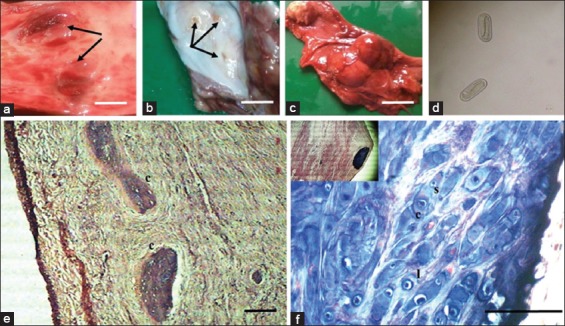
Gross and histopathology of *Spirocerca lupi*-infected esophagus (A) and (B) esophagus of *S. lupi*-infected dog showing multiple nodules in caudal esophagus (arrows). (C) Caudal part of esophagus of dog showing nodules protruding from the outer esophageal wall. (D) Typical capsule-shaped, thick-shelled larvated *S. lupi* egg 35 µm×15 µm (400×) presented in nodule. Bar=2 cm. (E) Cross-section of the distal esophageal nodule (c - chondrocyte tissue within the wall of the nodule). (F) Cross-section of distal esophageal wall showing anisokaryosis (variation in nuclear size) of the chondrocytes. (C) Different stages of cell division in the chondrocytes (s - small size nuclei and l - large size nuclei). Stained with Van Gieson stain and H and E. Bar=50 µm.

Of the infected animals, 33 (66.7%) were male and 21 (33%) were female dogs. Wide variety of dog breeds was encountered, and these included 29 (53.7%) cross-breeds, 16 (29.6%) German shepherd, 2 (3.7%) Dalmatian, 2 (3.7%) Ridgeback, 2 (3.7%) Rottweiler, and 3 (5.5%) Doberman were diagnosed with spirocercosis. In this study, the prevalence rate was higher in dogs over 1 year of age (78%) than dogs <1 year (22%). However, there was no statistically significant association between gender (p=0.228), age (p=0.568), and breeds (p>0.05) with the occurrence of spirocercosis. Moreover, all esophageal nodules identified were located near the caudal segment, and their diameters ranged from 1 to 6 cm (4.7±1.5 cm). A number of worms in each nodule varied from 5 to 25 (11.3±5). There were usually one to four worm nodules presented most commonly at the level of caudal esophagus (Figure-1A-D). Consistent with other reports, the results of the present study show no significant difference between the gender and age distributions with spirocercosis [7]. However, there seems to be a breed predilection [9]. As evident in the present study, cross-bred dog population represents >50% spirocercosis prevalence among five other large breeds, without significant association with occurrence of spirocercosis. It indicates fascinating phenomena of definite impact of different lifestyle of different breeds and exposure to the intermediate or paratenic host on disease transmission. Moreover, our cross-sectional study of necropsy findings showed 33% of the prevalence rate of spirocercosis among the dog population of the particular study area.

Histopathological examination of 15 formalin-fixed esophageal nodular sections revealed different stages of *S. lupi*-induced lesions in the caudal part of esophagus. Of the 15 histological sections, 11 were classified as non-neoplastic esophageal nodules while four had added changes reflecting dysplastic changes. The dysplastic sections contained with highly proliferative atypical chondroblasts in the wall of esophageal nodule as cell clusters associated with chondroid matrix. The chondrocyte’s clusters in some lesions contain chondrocytes with anisokaryosis (Figure-1E and F). In the non-neoplastic group, evidence of larval migration was detected in almost all cases; a single or many worm sections were present in 91% of cases while eggs were also noted in 45% of cases. The inflammatory infiltrates surrounding the parasite composed of neutrophils, small numbers of lymphocytes, eosinophils, fibroblasts, and the mature connective tissue, vascularized with small blood vessels forming the fibrovascular tissue in the nodule.

The association between spirocercosis and neoplasia was first reported in the 1950s [6] with numerous subsequent reports in the literature during the 1960s-1970s [1,9,10]. The incidence of esophageal fibrosarcoma and osteosarcoma is relatively high in *S. lupi* endemic areas [11]. Osteosarcoma appears to occur more commonly than fibrosarcoma, being present in approximately 60% of cases [9]. In a report from Kenya, 42 of 206 cases of *Spirocerca* had sarcomas, with 17/42 (40.5%) being fibrosarcomas and 25/42 (60%) osteosarcomas [12]. However, the association of chondrosarcoma with *S. lupi* is not frequently reported [13], and the pathogenesis of *S. lupi* neoplastic transformation is unclear, but two leading hypotheses are described to explain the infection-associated neoplastic transformation. First, uncontrolled local inflammation leading to genetic instabilities and malignant transformations [14] and second, the conversions are caused by the parasite itself, most likely in combination with the inflammatory response it produces [15,16].

Some of the limitations of our studies are the low number of samples and lack of detailed immunohistopathological identifications. To the author’s knowledge, no similar studies have previously been reported to determine a priori what the sample size of the current study should be. While we characterized chondrocyte’s clusters in some lesions contain chondrocytes with anisokaryosis, quantification and additional genomic analysis would have been more desirable, particularly from a statistical viewpoint.

## Conclusion

In this study, we identified and characterized canine *S. lupi*-induced esophageal chondrosarcoma by histopathology from the samples obtained at autopsy, whereas additional investigations will be required for detailed confirmation and clarify the mechanism of chondrosarcoma formation. However, there was no statistically significant association between gender, age, and breeds with the occurrence of spirocercosis. As the present study confirms potential of formation of esophageal chondrosarcoma through *S. lupi* infection, this finding might be used in designing the prophylactic and therapeutic strategies for managing the *S. lupi* suspected dogs.

## Authors’ Contributions

HMSW was responsible for the study concept, design, conducting all experiments, acquisition of data and all the data analysis, and drafting of the manuscript. DMSM, KANW, NH, and JR were involved in the design of study concept, drafting, and revising of the manuscript. HMHSA helped to collect and interpret the findings. DDNDS was a major contributor in conception and design of the study, interpretation of data, drafting, and revising the manuscript. All authors contributed to the interpretation of the data and approved the final manuscript.
